# Immune-modulating effects of bevacizumab in metastatic non-small-cell lung cancer patients

**DOI:** 10.1038/cddiscovery.2016.25

**Published:** 2016-10-03

**Authors:** EC Martino, G Misso, P Pastina, S Costantini, F Vanni, C Gandolfo, C Botta, F Capone, A Lombardi, L Pirtoli, P Tassone, C Ulivieri, P Tagliaferri, MG Cusi, M Caraglia, P Correale

**Affiliations:** 1 Radiotherapy Unit, Department of Oncology, Siena University Hospital, Siena, Italy; 2 Department of Biochemistry, Biophysics and General Pathology, Second Naples University, Naples, Italy; 3 CROM, Mercogliano, Italy; 4 Microbiology and Virology Unit, Department of Medical Biotechnology, Siena, Italy; 5 Medical Oncology Unit, ‘Magna Graecia’ University and AUO ‘Materdomini’, Catanzaro, Italy; 6 Department of Science of Life; University of Siena, Siena, Italy

## Abstract

The mPEBev is an anticancer regimen which combines a chemotherapy doublet, based on cisplatin and oral etoposide (mPE), with bevacizumab (mPEBev), a mAb targeting the vasculo-endothelial growth factor (VEGF). In previous studies, this regimen showed powerful anti-angiogenetic effects and significant antitumor activity in metastatic non-small-cell lung cancer (mNSCLC) patients. We also recorded the best benefit in patients exhibiting low-systemic inflammatory profile at baseline. On these bases, we hypothesized that mPEBev antitumor activity could be partially related to bevacizumab-associated immunological effects. For this reason, we performed an immunological monitoring in 59 out of 120 stage IIIb-IV NSCLC patients enrolled in the BEVA2007 phase II trial, who received fractioned cisplatin (30 mg/sqm days 1-3q21) and oral etoposide (50 mg, days 1-15q21) (mPE doublet) ±bevacizumab. In this group of patients, 12 received the mPE doublet alone and 47 the doublet in combination with bevacizumab (5 mg/kg on the day 3q21; mPEBev regimen). Blood cell counts, serum analysis, multiplex cytokine assay and immunocytofluorimetric analysis, performed on baseline and post-treatment on blood samples from these patients, revealed that bevacizumab addition to the doublet decreased levels of pro-angiogenic (VEGF, Angiostatin-1 and Follistatin) and inflammatory cytokines (interferon (IFN)γ, IL4 and IL17), improved *in vivo* and *in vitro* cytotoxic T-lymphocytes (CTL) response and promoted dendritic cell activation. These results suggest that the mPEBev regimen improve the micro-environmental conditions for an efficient antigen-specific CTL response, making it a feasible candidate regimen to be assessed in combination with immune-checkpoint inhibitors in NSCLC patients.

Non-small-cell lung cancer (NSCLC) is the most common malignancy and the leading cause of cancer death worldwide.^1^ The majority of NSCLC patients who cannot undergo curative surgery and who are diagnosed with advanced disease, have a poor prognosis with a survival time that usually does not exceed 8–10 months.^2^ The standard treatment for metastatic (m) NSCLC patients is based on doublets of platinum derivatives in combination with a second cytotoxic drug,^2,3^ or molecular target-specific inhibitors for patients presenting activating EGFR mutations (Erlotinib, Gefitinib and Afatinib) or EML-ALK translocations (Crizotinib, etc.).^4,5^ The efficacy of poly-chemotherapy in non-squamous NSCLC has been further improved by a multidrug combination with bevacizumab, a humanized IgG1 to the vascular endothelial growth factor (VEGF).^6,7^ More recently, active immunotherapy and immune-checkpoint inhibitors are entering in the treatment of mNSCLC. In particular, two monoclonal antibodies (mAbs), Nivolumab and Pembrolizumab, have shown evidence of antitumor activity in these patients.^8–13^ Nivolumab and Pembrolizumab are two mABs directed to the programmed cell death receptor (PD)1, commonly expressed on activated antigen-specific cytotoxic T lymphocytes (CTLs), residual of a pre-existing tumor-specific immune-response.^8–13^ PD1 binding with its specific ligands (PDL-1 and 2) in tumor tissue, leads to the immediate deactivation of the effector cells^8–13^ and, therefore, it represents a powerful inhibitory immune-checkpoint and a formidable mechanism of immune-escape for cancer cells.^8–13^ In this context, it has been shown that the VEGF deprivation induced by bevacizumab may stimulate immunological alterations, which could contribute to enhance the efficacy of chemotherapy and the survival of cancer patients.^14–18^ In fact, VEGF is a soluble dimeric protein family with multiple bio-regulative activities, mainly released in hypoxic and inflammatory conditions by mature granulocytes and platelets.^19–22^ It is worldwide known for its ability in inducing endothelial proliferation, neo-vessel formation and normalization in cancer patients; however, its bio-regulative activity is very pleyotropic and complex, and also involves the anticancer immune-system. In fact, its effects are mediated throughout the binding to five different membrane receptors, which are, in turn, expressed on endothelial precursors and other cell lineages including myeloid precursors, dendritic cells (DCs), lymphocytes and mesencephalic neurons.^19–22^ Therefore, VEGF release might exert multiple and different functions, including both neutrophils’ and inhibitory myeloid cells’ maturation, as well as inhibitory effects on DC maturation and CTL precursors’ activation.^14–18^ We have previously designed a phase I/II clinical trial (BEVA 2007 study) aimed to investigate the toxicity and the biological and antitumor activity of a novel metronomic bio-chemotherapy regimen (mPEBev) in mNSCLC patients. This regimen combined a previously described mPE doublet of cisplatin and oral etoposide, with bevacizumab (Bev). Our preliminary results showed that the addition of bevacizumab to the metronomic doublet was safe and very active in term of antitumor activity.^23–26^ We also found that the mPEBev administration was followed by a rapid decline in the primary tumor blood flux (perfusional CT scan)^25^ paralleled by a significant decline in VEGF, angiopoietin-1, thrombospondin-1 serum levels^25^ and systemic inflammatory markers (NLR, CRP, LDH and myeloperoxidase).^23–26^ An additional study carried out by our group, demonstrated that the best advantage on progression-free survival and overall survival were recorded in patients who presented a lower systemic inflammatory profile before the beginning of the treatment.^27^ These findings suggested that bevacizumab and, therefore, VEGF deprivation could synergize with the cytotoxic drug doublet through different mechanisms: (i) direct anticancer effects, (ii) anti-angiogenetic activity and (iii) immune-modulating activity. On these bases, with the present study we have evaluated immunological alterations in serum and peripheral blood mononuclear cells (PBMCs) derived from 59 patients who had received mPE doublet ±bevacizumab from September 2012 to May 2015. We also performed a fuctional *ex vivo* study on antigen-specific T-cell lines generated from patients’ PBMCs isolated at baseline and after two and four treatment courses.

## Results

### Demography

The BEVA2007 was a phase II trial, designed on translational bases and approved by the Siena University Ethical committee on March 2007. It was performed to assess toxicity, biological activity and antitumor activity of the mPE doublet ±bevacizumab in mNSCLC patients. One-hundred and fifteen, stage IIIb-IV NSCLC, patients signed an informant consent and were enrolled in the BEVA2007 trial since April 2007. All of them received the chemotherapy doublet, alone (30 patients; PE group) or combined with bevacizumab (85 patients; mPEBev group). We subsequently performed a pre-ordered immune-biological investigation, on the peripheral blood of 59 (51%) of these patients. Twelve out 59 patients had received the mPE doublet alone, whereas 47 had received the doublet combined with bevacizumab (mPEBev). Blood cell counts, serum analysis, multiplex assays and flow cytometry analysis were performed on blood samples isolated at baseline and after two and four treatment courses. The clinical features of these patients are shown in the Table 1.

### Pro-angiogenic factors

**Figure 1:**
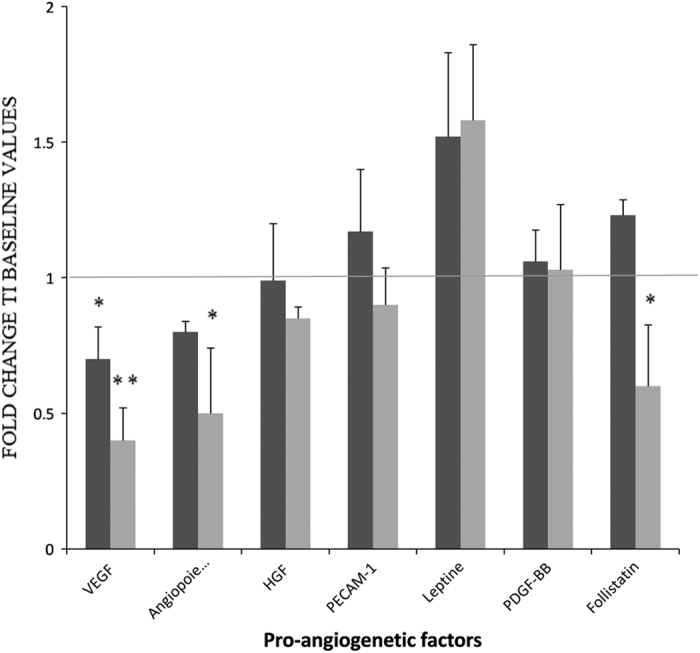
A multiplex analysis concerning the dosage of multiple pro-angiogenetic factors in the serum of mNSCLC patients enrolled in the BEVA2007 trial who have received the mPE doublet alone (
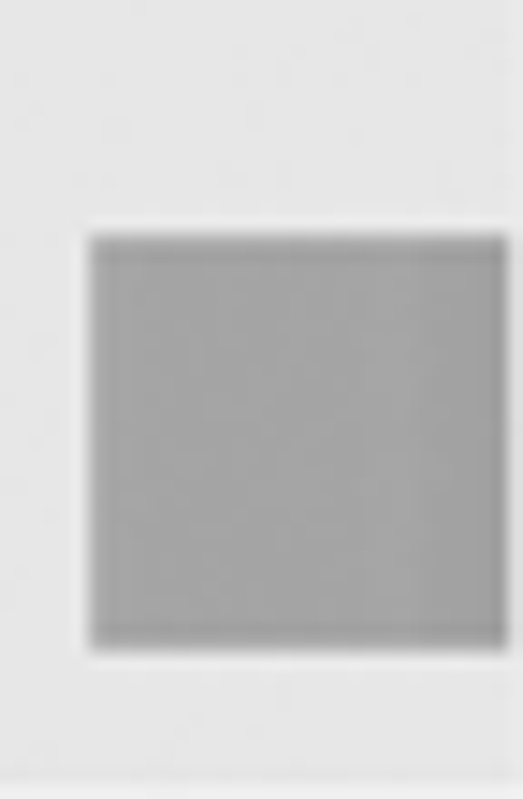
) or combined with bevacizumab (
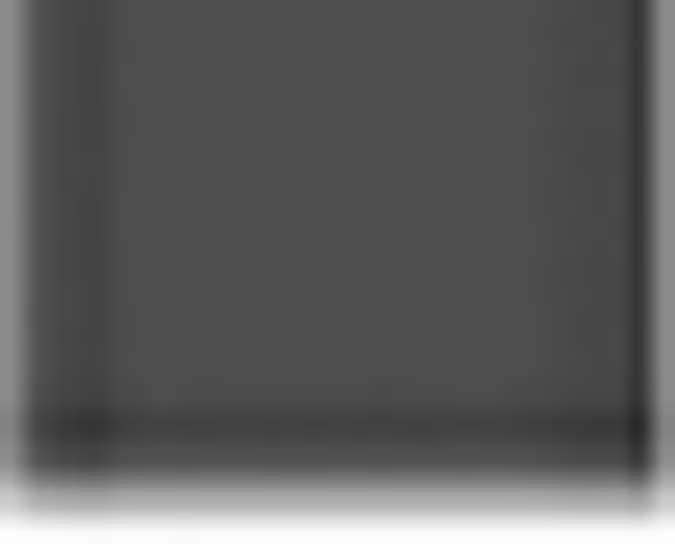
). Results are expressed as fold induction relative to baseline indicated as 1 (±S.E.). Asterisks represent statistical significance to the correspondent baseline values (*P<0.05; **P<0.01).

Our multiplex assays detected a significant decline in the serum levels of multiple pro-angiogenic factors in the group of patients who received the mPEBev regimen, where it was found a significant decrease in VEGF (early event), angiopoietin-2 and follistatin (late events). For these patients, we were unable to demonstrate any significant change in the levels of other factors potentially involved in both tumorigenesis and neo-angiogenesis, such as HGF, PECAM-1, Leptin, PDGF-BB, G-CSF and IL8 (Figure 1).

### Inflammatory cytokine release

**Figure 2:**
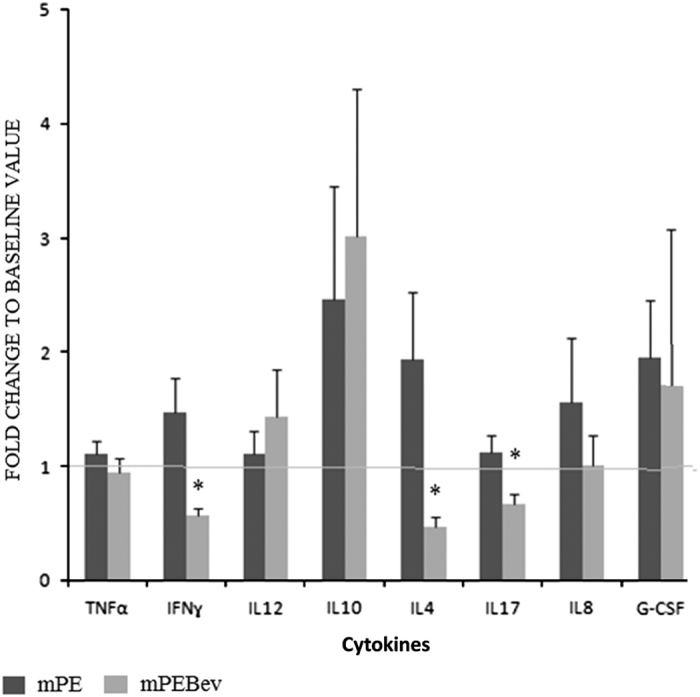
A multiplex analysis concerning the dosage of T_h_1 (IFNɣ, TNF*α* and IL12), T_h_2 (IL4 and IL10), T_h_17 and inflammatory cytokines (IL8 and G-CSF) in the serum of mNSCLC patients enrolled in the BEVA2007 trial who have received the mPE doublet alone (
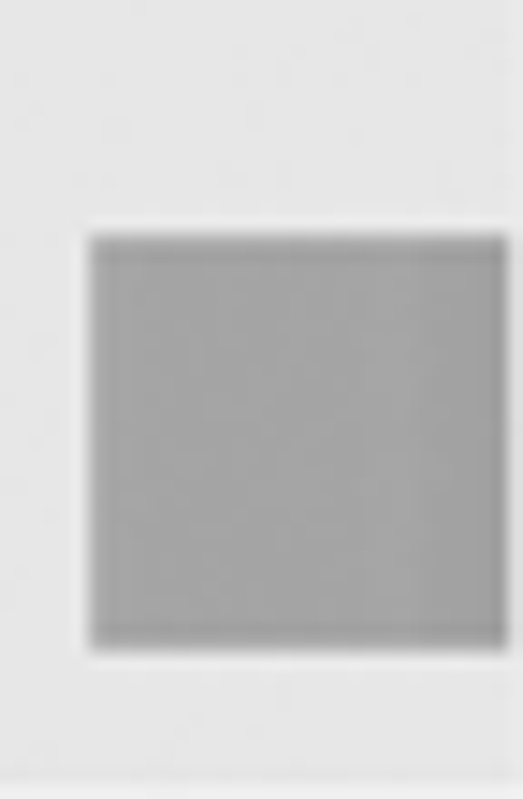
) or combined with bevacizumab (
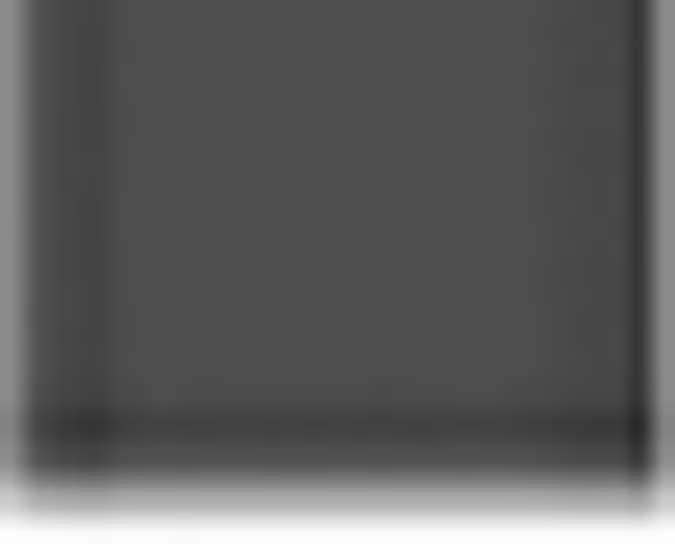
). Results are expressed as fold induction relative to baseline indicated as 1 (±S.E.). Asterisk represents statistical significance to the correspondent baseline values (*P<0.05).

We performed, in the same patients, an additional multiplex assay to detect possible treatment-associated changes in serum levels of cytokines involved in different immunological and inflammatory profiles. We found a significant decrease of IFNγ (T_h_1), IL4 (T_h_2) and IL17 (T_h_17) levels only in the group of patients who received the bevacizumab-based treatment and not in those who received the mPE doublet. The latter group of patients, conversely, showed a trend to increase IL8 and IFNγ levels, even though without reaching statistical significance. Finally, both groups of patients showed a trend to increase IL10 and G-CSF levels, also in this case,without reaching statistical significance (Figure 2).

### Immunocytofluorimetric analysis

**Figure 3:**
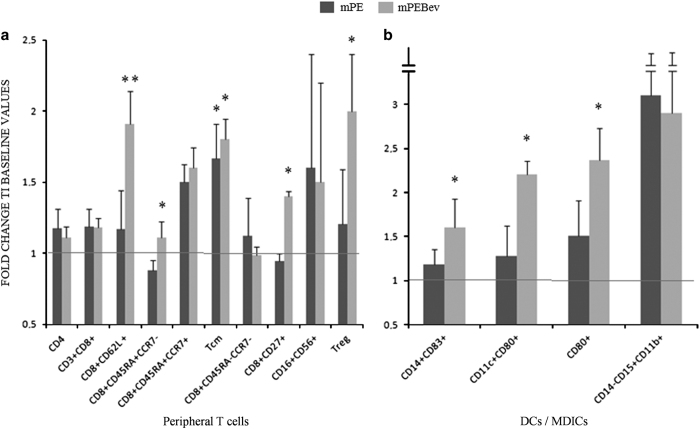
A flow cytometric analysis concerning the expression of (**a**) different T-cell subsets and (**b**) myeloid derivative cells (activated DCs and MDICs) in the PBMCs of mNSCLC patients enrolled in the BEVA2007 trial who have received the mPE doublet alone (
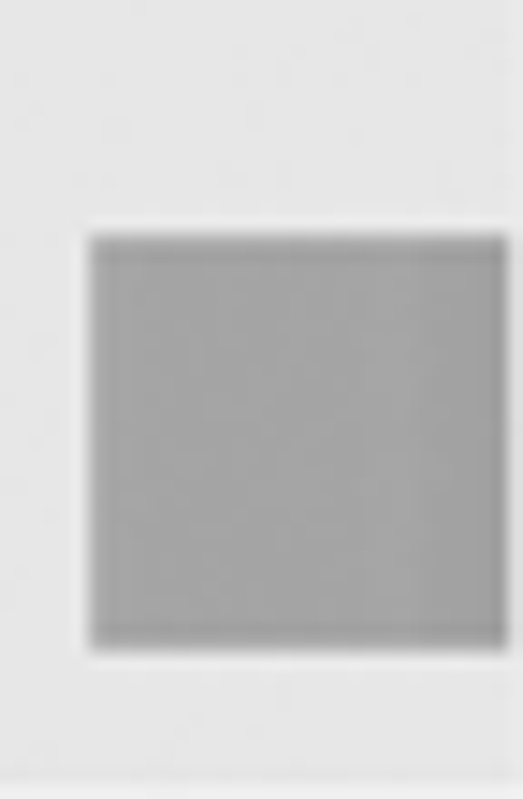
) or combined with bevacizumab (
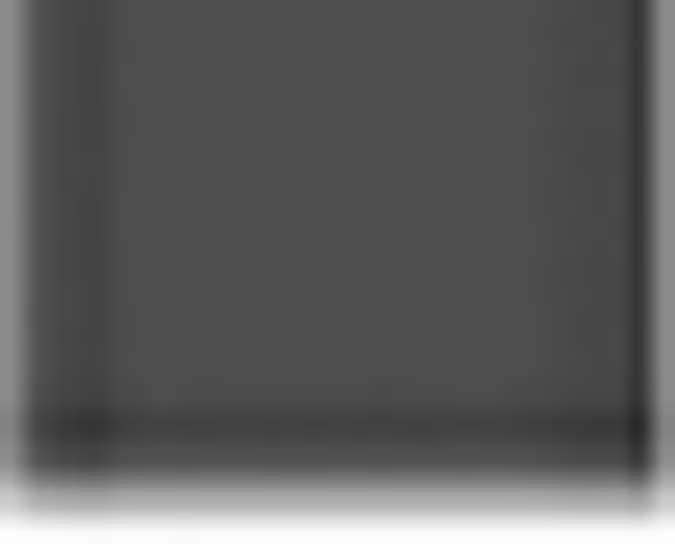
). Results are expressed as fold induction relative to baseline indicated as 1 (±S.E.). Asterisks represent statistical significance to the correspondent baseline values (*P<0.05; **P<0.01).

A multicolour flow cytometry study was carried out on the PBMCs from these patients, showing a parallel and coordinate increase in activated (CD8^+^CD62L^+^), central-memory (CD8^+^C45RA^−^CCR7^+^; T_cm_) and long-term memory (CD8^+^C45RA^−^CCR7^+^CD27^+^) CTLs; a trend to increase regulatory T cells (T_reg_) was recorded only in patients who received the mPEBev regimen. On the other hand, patients who received the metronomic doublet alone; showed a significant increase in T_cm_s (Figure 3a). In this set of patients, both treatment regimens did not induce significant changes in the levels of CD3^+^CD4^+^, CD3^+^CD8^+^ and natural killer cells (CD3^-^CD16^+^CD56^+^;NK) cells (*P*>0.05).

**Figure 4:**
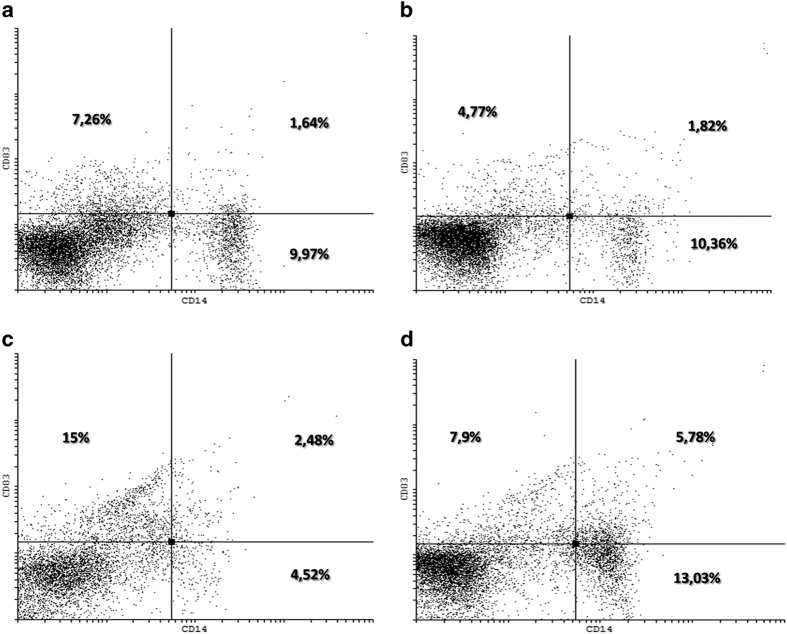
Flow cytometry dot plots of two representative patient PBMCs: activated DCs, isolated at the baseline (**a** and **b**) and after four treatment courses with mPE doublet and bevacizumab (**c** and **d**).

In PBMcs we also evaluated the effects of treatments on professional antigen-presenting cells, like DCs and myeloid derivative inhibitory cells (MDICs). We found that only mPEBev treatment, was associated with a significant increase in the percentage of activated DCs (CD3^−^CD19^−^CD11c^+^CD14^+^CD15^−^), expressing either CD83 or CD80. Conversely, an increasing trend, even if not significant, was observed in the percentage of activated MDICs (CD11b^+^CD14^+^CD15^+^) in both groups of patients (Figures 3b and 4).

### *Ex vivo* characterization of patients-derived antigen-specific T-cell lines

We performed an *ex vivo* study on PBMCs isolated from patients at the baseline and after four treatment courses with mPE (four patients) or mPEBev (four patients), to verify whether these regimens were able to interfere with the generation of active antigen-specific CTLs. We evaluated multiple T-cell functions which could be potentially affected by the metronomic doublet ±bevacizumab. Thus we cyclically stimulated ex *vivo* PBMCs collected from patients with three antigens with completely different features: (i) streptococcal-B-superantigen (SEB), a bacterial antigen able to over-stimulate both CD3^+^CD8^+^ and CD3^+^CD4^+^ subsets;^28^ (ii) immune-reconstituted influenza virosome (IRIV), an antigenic influenza virus membrane envelope, expressing high-immunogenic flow antigens and multiple T helper epitopes^29^ and (iii) thymidylate synthase poly-epitope peptide vaccine (TSPP), a 27-mer peptide which assembles multiple class I-HLA restricted CTL epitopes of the thymidylate synthase, a tumor-associated enzyme commonly overexpressed in colo-rectal and NSCLC cancer patients.^30^ TSPP was tested in previous preclinical studies showing the ability of generate an efficient antitumor *in vitro* CTL response*,* both in mice and in cancer patients.^31,32^

**Figure 5:**
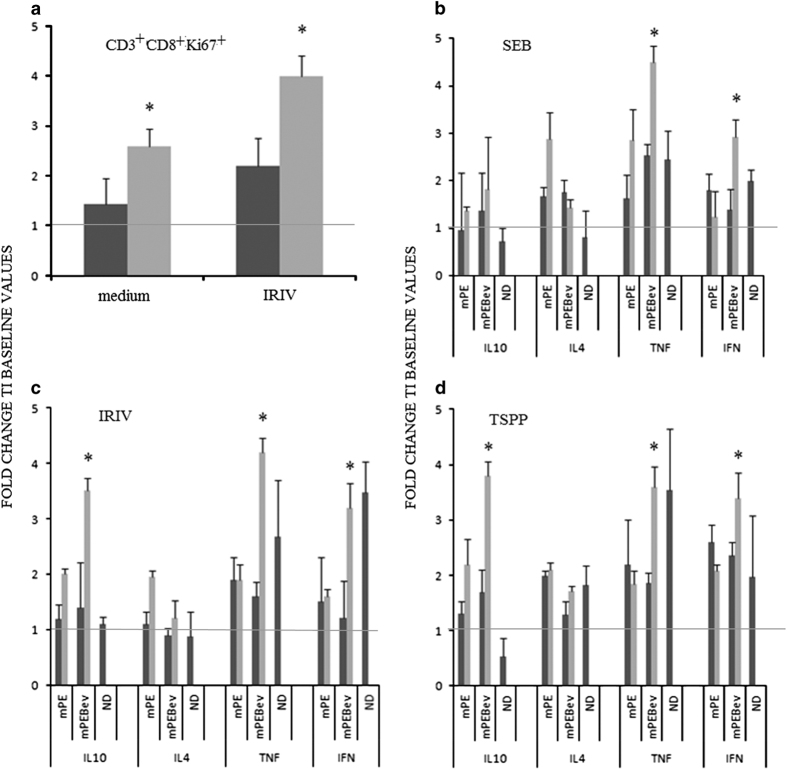
(**a**) A flow cytometric analysis concerning the expression of CD8^+^Ki67^+^ cells in T-cell cultures undergone multiple *in vitro* stimulation with IRIV and derived from the PBMCs of normal donors (ND) or mNSCLC patients isolated at the baseline (
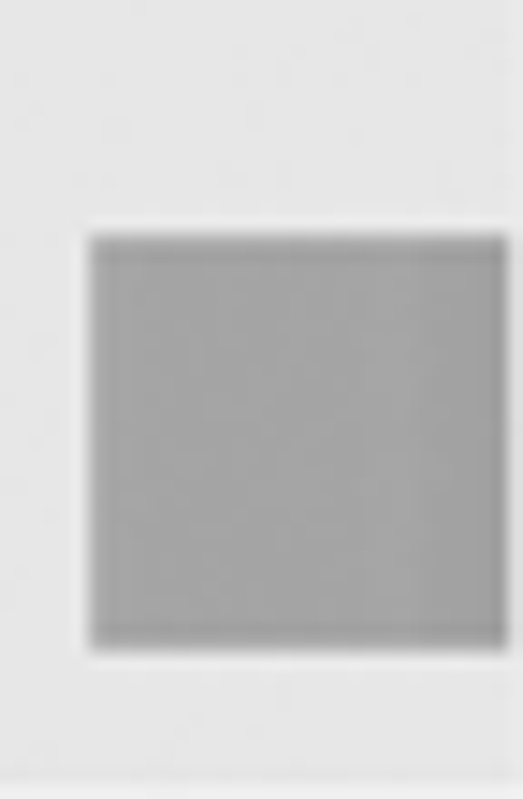
) and after four treatment courses (
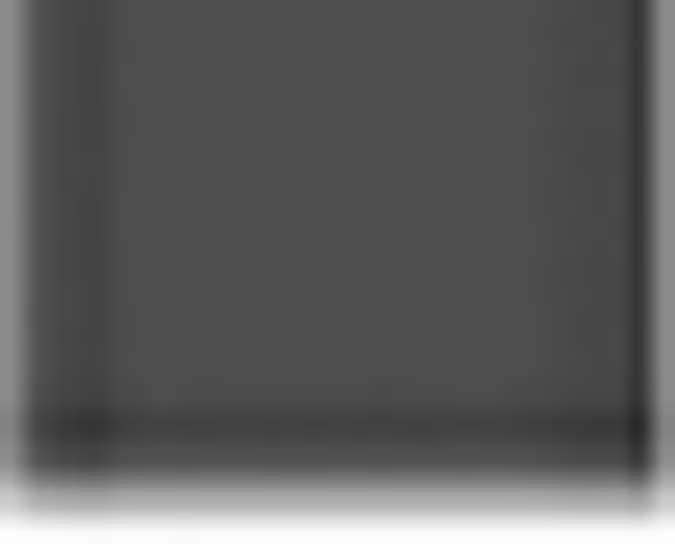
) with the mPE doublet alone or combined with bevacizumab (mPEBev). Further panels describe the ELISA dosage of T_h_1 (IFNɣ, TNF*α* ) and T_h_2 (IL4 and IL10) cytokines in the supernatant of T-cell cultures *in vitro* stimulated with SEB (**b**), IRIV (**c**) and TSPP (**d**), and derived from the PBMCs of normal donors (ND) or mNSCLC patients and isolated at the baseline (
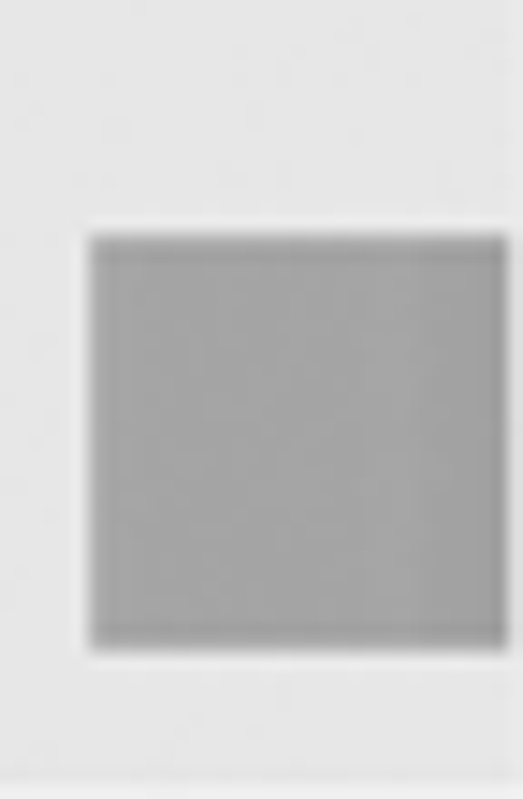
) or after four treatment courses (
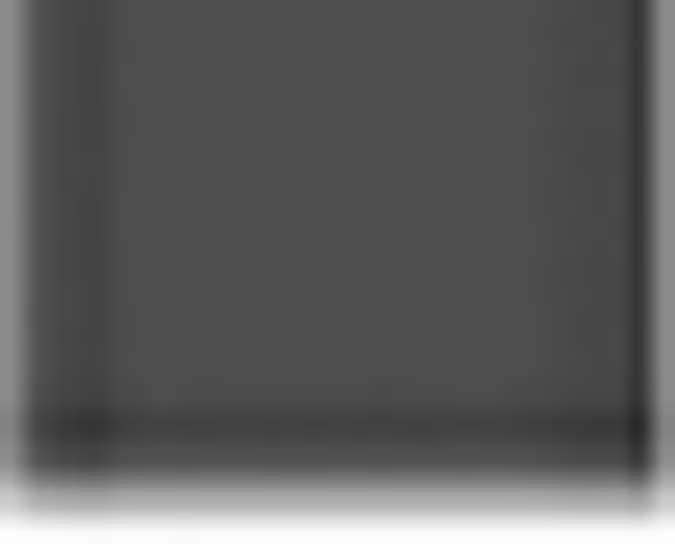
) with the doublet alone (mPE) or combined with bevacizumab (mPEBev). Results are expressed as fold induction relative to baseline indicated as 1 (±S.E.). Asterisk represents statistical significance to the correspondent baseline values (*P<0.05).

After multiple *in vitro* stimulations (IVSs), we evaluated the percentage of proliferative T cells (CD3^+^CD8^+^Ki67^+^) and Th1/Th2 cytokine release in response to the three antigens. In our setting, the T-cell lines derived from PBMCs isolated at the baseline, showed a minimal proliferative response to the three antigens (data not shown and Figure 5a). On the other hand, T-cell lines derived from PBMCs isolated after four treatments courses, showed a minimal proliferative response to TSPP and SEB (data not shown) and a higher response to IRIV (Figure 5a). Moreover, we observed a greater percentage of proliferative antigen-specific precursors in T-cell lines derived from patients receiving the mPEBev regimen, compared with the others (fold change to baseline values, mPE *versus* mPEBev=2.2 (±0,55) *versus* 4. 1 (±0,4), *P*=0.049; Figure 5a). The functional antigen-specific cytokine response of these T-cell lines also showed a powerful T_h_1 response with parallel tumor necrosis factor (TNF)*α* and IFNγ release, only in T cells derived from both mPEBev-treated patients and normal donors. Furthermore, there was no different response to the three antigens used for each specific T-cell line *in vitro* sensitization (Figures 5b–d). These experiments also showed that T cells derived from the mPEBev-treated patients had a greater IL10 release in response to IRIV and TSPP. On the other hand, patients receiving the chemotherapy doublet alone, prevalently showed a non-cytotoxic T_h_2 immune-response with an increased antigen-specific release of IL4 (Figures 5b–d).

## Discussion

In the present manuscript, we report the results of an immunological study performed on 59 patients, enrolled in the BEVA2007 trial, who received frontline treatment according to the mPE doublet ±bevacizumab. We show that the addition of the mAb to the chemotherapy in mNSCLC patients, as partially observed in our previous studies,^23–26^ decreases their systemic inflammatory status and promotes anticancer immune-modulating effects. In fact, we found that the mPEBev regimen causes, in patient's serum, significant reduction in the levels of VEGF and other anti-angiogenetic factors, including Angiopietin 2 and Follistatin, an inhibitor of both TGF-*β* superfamily activin and FGF-2R angiogenesis inducer.^33^ These events were paralleled by a progressive decline in NLR (data not shown),^26^ and IFNγ, IL4 and IL17 serum levels. All together these cytokines are expression of cancer-associated systemic inflammation, that is paralleled by immune-suppression and neo-angiogenesis;^26,34–38^ therefore, it is possible to hypothesize that a parallel and progressive reduction of pro-angiogenic factors can have a role in the ultimate antitumor effects of the mPEBev regimen. Bevacizumab is commonly considered an anti-angiogenic agent because it subtracts free VEGF that, in turn, promotes endothelial precursors’ recruitment and neo-angiogenesis in tumor tissues. However, it has to be considered that VEGF effects are not limited to endothelial cells. In fact, VEGF is not produced only by tumor, but it is also transported to tumor tissues by platelets and inflammatory cells (i.e.: neutrophils and monocytes) during cancer-associated inflammation and hypoxic status.^18–22^ VEGF, in addition, promotes activation and differentiation of neutrophils, monocytes and MDICs, and impairs the immune-system by affecting DCs and specific T-cell subsets.^18,22^ These findings support the hypothesis that a systemic inflammation state at the baseline (before the beginning of treatment) is strongly predictive of poor prognosis in cancer patients receiving bevacizumab and/or other immunological-based treatments. Moreover, it has also been reported that IL17, a pro-inflammatory cytokine produced by T_h_17 subsets upon stimulation by IFNγ, promotes immune-suppressive chronic inflammation that can be present in both auto-immune diseases and cancer. In fact, IL17 attracts neutrophils, myeloid derivative inhibitory cells and increases VEGF levels in tumor micro-environment. High IL17 levels are strictly involved in tumorigenesis, metastatization and in promotion of VEGF-independent neo-angiogenesis in different malignant diseases. In the case of lung cancer development, IL17 serum levels correlate to poor prognosis and serum VEGF levels.^37^ Moreover, IL17 promotes angiogenesis by stimulating VEGF production of NSCLC cells via the STAT3/GIV signaling pathway.^38^ The results of our study also revealed that bevacizumab combination with the anti-blastic doublet is correlated with a significant increase in activated (CD8^+^CD62L^+^) CTLs, long-term effector memory (CD8^+^CD27^+^) and central-memory (CD8^+^C45RA^-^CCR7^+^) CTLs. In addition, our *ex vivo* investigation was on line with these findings; in fact, antigen-specific T-cell proliferation, as well as a greater antigen-specific Th1 response with potential cytotoxic activity, was recorded only in PBMCs derived from patients who had received the mPE doublet plus bevacizumab. On the other hand, the homologs T cells lines derived from mPE group showed a poor antigen-specific proliferative response and a non-cytotoxic, pro-humoral Th_2_ response. In our experiments, the antigen-specific IL10 production suggests an immune-response as a possible inhibitory feed-back response to antigen-specific over-stimulation.^36^ In the present study, we were unable to identify possible effects of mPEBev on MDICs expression in patients’ blood samples; on the contrary, mPEBev regimen was able to increase the percentage of activated and mature myeloid derived DCs, as shown by a significant increase in CD83^+^ and CD80^+^-positive cells. The latter findings suggest that VEGF deprivation can improve antigen presentation by promoting DC expression and activation; this, in turn, can induce the expansion of the effector T-cell compartment with long-term memory and high-tumor-specific killing activity and enable to achieve distant lymph-nodes and tumor sites, producing the chemotactic chemokines 19 and 21 recognized by the chemokine receptor (CCR)-7 expressed on the surface of these CTLs.^39,40^

It is commonly accepted that in condition of hypoxia and chronic inflammation, granulocyte produce high levels of VEGF that, in turn, by interacting with the FLT-1/VEGFR1, promotes neo-angiogenesis for tissue repair, and the expansion and maturation of additional myeloid cells.^41,42^ MDICs family is a blood cell lineage population dependent by VEGF, IL10 and IL-13 levels. Once activated, MDICs may exerts a powerful immunological inhibitory activity affecting the activation of both DCs’ and antigen-specific CTLs’ precursors.^41,42^ On this light, we have previously reported that VEGF deprivation, induced by the mPEBev regimen, is associated to a progressive decrease in the number and maturation of neutrophils.^41,42^ In the present study, we were unable to demonstrate possible treatment-associated changes in the percentage of CD14^−^CD15^+^CD11b^+^CD66b^+^ MDICs in patients' peripheral blood, mostly due to the significant heterogeneous results obtained in these patients' samples. However, the present finding do not exclude that the inhibitory activity of these cells is repressed by VEGF deprivation, as suggested by the results on DC activation, CTL response and *ex vivo* results on antigen-specific CTLs. Additional experiments are presently ongoing to evaluate the effective role of MDICs deactivation in patients receiving bevacizumab and platinum based doublets. In previous studies, in line with the results of other authors, we have observed that patients receiving mPEBev treatment may develop parenchimal fibrosis and cavitations in lung sites apparently not involved by the tumor and more rarely in lethargic encephalitis.^23–26,43,44^ These events can be, at least in part, explained by the occurrence of autoimmunity or of excessive immune-effector-mediated response to subclinical viral infections.^43,44^ On the basis of these results, we can hypothesize that the immunological effects associated to bevacizumab administration could, at least theoretically, synergize with chemotherapy in enhancing the final antitumor effect in mNSCLC patients. In this light, it has been proposed that necrosis, massive antigen release and the formation of apoptotic bodies, caused by cytotoxic drugs in tumor tissues, could decrease the levels of immune-response inhibition mediated by PD-1/PD-1 ligand interactions. The latter effect can, in turn, cause a powerful immunological danger signal, thus leading to an increase of efficient antigen-specific CTL response with long-term memory. This observation has represented the bases for the combination of chemotherapy and/or radiotherapy with immune-adjuvant cytokines, active-specific immunotherapy and checkpoint inhibitors, in the treatment of human malignancies.^44–51^ Previous results from us and others have already shown preclinical evidence that both chemotherapy and bevacizumab administration may affect MDICs and T_reg_s’ expansion,^50,51^ whereas the present study demonstrate that it may also improve DC maturation and enhance CTL response, thus producing a more complex and efficacious antitumor effect. Moreover, the long-term effects of bevacizumab on the effector memory T cells and T_h_1 lymphocyte subsets could have a positive impact on patients’ survival because the antitumor specific immune-response can be auto-sustained after the end of the treatment.^50,51^ In conclusion, bevacizumab immune-modulating effects, together with its anti-angiogenetic properties, could equally contribute to define the ultimate antitumor effects in cancer patients, thus increasing their survival. Our results suggest that the mPEBev regimen promotes the best conditions for an efficient antigen-specific CTL response and makes the mPEBev a potential candidate regimen to be assessed in combination with immune-checkpoint inhibitors in NSCLC patients.

## Subjects and methods

### Study design

The study protocol code #BEVA2007 was performed in accordance to the good clinical practice guidelines and was approved by the Bioethics Committee of the University of Siena. All patients provided a written informed consent. The inclusion criteria were: histological diagnosis of mNSCLC, performance status (ECOG) from 0 to 2, normal renal and hepatic function, WBC count >2500/mm^3^, hemoglobin >9 g/dl, platelet cell count >90 000/mm^3^ and normal cardiac function. The exclusion criteria were: central tumors with high risk of bleeding (excavated with large necrosis and infiltration of large arterial and venous structures) for bevacizumab use, a history of other severe cardiovascular disease, arrhythmia, second malignant tumors and signs of active infections.

### Treatment schedule

All of the patients received every 3 weeks, iv. cisplatinum (30 mg/sqm) on days 1-3, daily oral etoposide (50 mg) on days 1–15 and bevacizumab at 5 mg/kg on the day 3, for a maximum of four consecutive cycles. Subsequently, all patients who did not show a progression of disease, received erlotinib administration (150 mg/die) until progression of disease, starting 1 week after last chemotherapy course.

### Biological analysis and blood sampling

Peripheral blood samples (10 ml) were withdrawn at baseline and 1 h before any treatment cycle, for either serum and PBMC isolation. Serum derived from standard peripheral blood centrifugation and PBMCs obtained by Ficoll-Hypaque (Celbio S.P.A., Italy) gradient separation medium, form heparinized blood samples, were immediately frozen and stored as described in previous studies.^52^ Lymphocytes, platelets, neutrophils and monocytes were evaluated by hemocytometric cell counts, while their feature was evaluated by microscope analysis. Flow cytometry was perfomed on patients’ PBMCs by carrying out standard multicolor immuno-cytoflurimetric analysis with conjugated anti-CD3, CD4, CD8, CD27, CD62L, CD19, CD16, CD56, CD25, FoxP3, CCR7, CD45Ra, CD11b, CD11c, CD14 and CD15, all purchased by eBioscience, USA.

### Bio-Plex assay

Blood samples were collected from a peripheral vein at baseline and after 3 treatment courses and kept on ice. Serum was collected by centrifugation (3000 r.p.m. for 10 min at 4 °C), aliquoted and stored at −80 °C until analyzed. A multiplex biometric ELISA-based immunoassay, containing dyed microspheres conjugated with a monoclonal antibody specific for a target protein was used according to the manufacturer’s instructions (Bioplex, Bio-Rad Lab., Inc., Hercules, CA, USA). Soluble molecules were measured using either commercially available kits or customized kits for the evaluation of the following cytokines: Interleukin(IL)4, IL8, IL10, IL12, IL17, IFNγ, TNF*α*, VEGF, granulocyte colony-stimulating factor, angiopoietin-2, HGF, PECAM-1, Leptin, PDGF.BB and Follistatin. Each experiment was performed in duplicate using the same procedure described in previous papers.^53^ Serum levels of all proteins were determined using a Bio-Plex array reader (Luminex, Austin, TX, USA) that quantifies multiplex immunoassays in a 96-well plate with very small fluid volumes. The analyte concentration was calculated using a standard curve, with software provided by the manufacturer (Bio-Plex Manager Software, Hercules, CA, USA).

### *In vitro* stimulation of patients’ PBMC

PBMCs were isolated from four different patients who had received the mPEBev regimen and three patients who had received the mPE treatment. Blood samples taken at baseline and after three treatment courses were seeded in 24-multi-well plate at the final concentration of 10^6^ cells/ml in complete medium (AIM-V) with 5% heat inactivated human AB serum. As previously described,^30^ PBMCs were *in vitro* stimulated every 15 days with autologous irradiated PBMCs loaded with SEB (1 *μ*g/ml), IRIV (5 *μ*g/ml) or TSPP (10 *μ*g/ml), in complete medium containing IL4 (0.5 ng/ml), granulocyte macrophage colony-stimulating factor (15 ng/ml) for 5 days, and then followed by 10 days of culture in the presence of IL2 (25 UI/ml) before being restimulated with the same modalities.

Flow cytometry was performed on T-cell lines after three IVS with IRIV, whereas cytokine assays were performed by Bioplex analysis on the supernatant T cells after 4 IVS with the specific antigen.

### Statistical analysis

The between-mean differences were statistically analyzed using Stat View statistical software (Abacus Concepts, Berkeley, CA). The results were expressed as the mean±S.D. of four determinations made in three different experiments, and the differences determined using the two-tail Student’s *t*-test for paired samples. A *P*-value of 0.05 or less was considered statistically significant.

## Figures and Tables

**Table 1:** Patients enrolled in the BEVA2007 trial undergone the immune-biological monitoring

	*mPE*	*mPEBev*
Patient number	12	47
Age	69 (range 59–83)	62 (range 33–82)
		
*Gender*		
Male	10	37
Female	2	10
		
*Hystology*		
Adenocarcinoma	5	32
Squamous	4	6
Undifferentiated	2	4
Not specified	1	4
